# 3,3-Dinitro­azetidinium chloride

**DOI:** 10.1107/S1600536812046302

**Published:** 2012-11-17

**Authors:** Biao Yan, Hong-Ya Li, Ning-Ning Zhao, Jie Li, Hai-Xia Ma

**Affiliations:** a School of Chemistry and Chemical Engineering, Yulin University, Yulin 719000 Shaanxi, People’s Republic of China; b School of Chemical Engineering, Northwest University, Xi’an 710069 Shaanxi, People’s Republic of China

## Abstract

In the title *gem*-dinitro­azetidinium chloride salt, C_3_H_6_N_3_O_4_^+^·Cl^−^, the cations and anions lie on a mirror plane. The azetidine ring is virtually planar, with a mean deviation from the plane of 0.0569 Å. The dihedral angle between the two nitro groups is 90.00 (5)°. In the crystal, the ions are linked by N—H⋯Cl interactions, forming a chain along the *c*-axis direction, and C—H⋯O inter­actions, forming a layer parallel to (010).

## Related literature

For 1,3,3-trinitro­azetidine and compounds prepared from its derivative 3,3-dinitro­azetidine, see: Archibald *et al.* (1990 ▶); Hiskey *et al.* (1992 ▶); Ma *et al.* (2009*a* ▶,*b* ▶, 2011 ▶); Yan *et al.* (2009 ▶, 2010 ▶); Gao *et al.* (2009 ▶). For related structures, see: Gao *et al.* (2010 ▶); Ma *et al.* (2010 ▶). For the synthesis, see: Li *et al.* (2004 ▶).
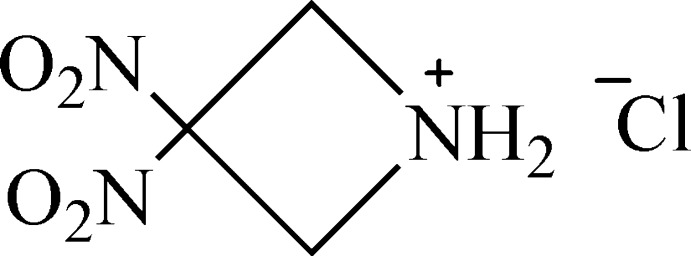


## Experimental

### 

#### Crystal data

C_3_H_6_N_3_O_4_^+^·Cl^−^
*M* *_r_* = 183.56
Orthorhombic, 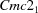

*a* = 6.6807 (17) Å
*b* = 10.4409 (17) Å
*c* = 9.9707 (19) Å
*V* = 695.5 (2) Å^3^
*Z* = 4
Mo *K*α radiation
μ = 0.52 mm^−1^
*T* = 293 K
0.35 × 0.34 × 0.30 mm

#### Data collection

Bruker SMART APEXII CCD area-detector diffractometer
Absorption correction: multi-scan (*SADABS*; Sheldrick, 2000 ▶) *T*_min_ = 0.839, *T*_max_ = 0.860
1968 measured reflections
708 independent reflections
696 reflections with *I* > 2σ(*I*)
*R*_int_ = 0.019

#### Refinement

*R*[*F* ^2^ > 2σ(*F* ^2^)] = 0.021
*wR*(*F* ^2^) = 0.055
*S* = 1.10
708 reflections
62 parameters
1 restraint
H-atom parameters constrained
Δρ_max_ = 0.18 e Å^−3^
Δρ_min_ = −0.16 e Å^−3^
Absolute structure: Flack (1983 ▶), 252 Friedel pairs
Flack parameter: 0.09 (7)

### 

Data collection: *APEX2* (Bruker, 2003 ▶); cell refinement: *SAINT* (Bruker, 2003 ▶); data reduction: *SAINT*; program(s) used to solve structure: *SHELXS97* (Sheldrick, 2008 ▶); program(s) used to refine structure: *SHELXL97* (Sheldrick, 2008 ▶); molecular graphics: *SHELXTL* (Sheldrick, 2008 ▶); software used to prepare material for publication: *SHELXTL*.

## Supplementary Material

Crystal structure: contains datablock(s) I, global. DOI: 10.1107/S1600536812046302/zq2187sup1.cif

Structure factors: contains datablock(s) I. DOI: 10.1107/S1600536812046302/zq2187Isup2.hkl

Supplementary material file. DOI: 10.1107/S1600536812046302/zq2187Isup3.cml

Additional supplementary materials:  crystallographic information; 3D view; checkCIF report

## Figures and Tables

**Table 1:** Hydrogen-bond geometry (Å, °)

*D*—H⋯*A*	*D*—H	H⋯*A*	*D*⋯*A*	*D*—H⋯*A*
N1—H1*C*⋯Cl	0.90	2.35	3.087 (2)	139
N1—H1*D*⋯Cl^i^	0.90	2.19	3.0575 (19)	163
C1—H1*B*⋯O4^ii^	0.97	2.58	3.543 (2)	172

Symmetry codes: (i) 
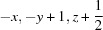
; (ii) 
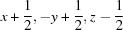
.

**Fig. 1.**
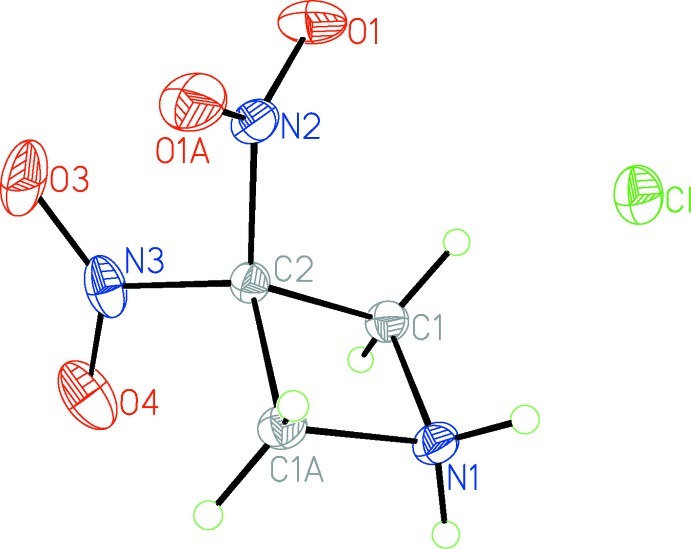
The molecular structure of the title compound, showing the atom-numbering scheme. Displacement ellipsoids are drawn at the 30% probability level and H atoms are drawn as spheres of arbitrary radius.

## supplementary crystallographic information

### Comment

Dinitro- and trinitro-derivatives of azetidine are of interest because they contain strained ring systems. This makes them good candidates for energetic materials (propellants or explosives). Azetidine-based explosives, such as 1,3,3-trinitroazetidine (TNAZ) (Archibald *et al.*, 1990) demonstrate excellent performance partly because of the high strain associated with the four-membered ring. As one of the important derivates of TNAZ, 3,3-dinitroazetidine (DNAZ) (Hiskey *et al.*, 1992) can prepare a variety of solid energetic materials with high oxygen-balance (Ma *et al.*, 2009*a*; Ma *et al.*, 2009*b*; Yan *et al.*, 2009; Gao *et al.*, 2009; Yan *et al.*, 2010; Ma *et al.*, 2010; Gao *et al.*, 2010; Ma *et al.*, 2011). This paper reports the crystal structure of the title DNAZ salt, C_3_H_6_N_3_O_4_^+^.Cl^-^.

In the title dinitroazetidinium chloride salt, cations and anions lie on a mirror plane. The azetidine ring is virtually planar, with a mean deviation from the plane of 0.0569 Å. The dihedral angle between the two nitro groups is 90.00 (5)°. In the crystal, the ions are linked by N–H···Cl and C–H···O interactions.

### Experimental

The title compound was synthesized and purified by a reported method (Li *et al.*, 2004). The compound was then dissolved in water and colorless crystals were isolated after 1 d.

Elemental analysis calculated for C_3_H_6_N_3_O_4_Cl: C 19.63, N 22.89, H 3.29%; found: C 19.74, N 23.10, H 3.19%.

IR (KBr, cm^-1^): 3057, 2623, 1588, 1406, 1333, 850, 808.

### Refinement

H atoms were placed at calculated idealized positions and refined using a riding model, with C—H = 0.97 Å and N—H = 0.90 Å [and *U*_iso_(H) = 1.2*U*_eq_(C,N)].

### Crystal data

**Table:**

C_3_H_6_N_3_O_4_^+^·Cl^−^	*Z* = 4
*M**_r_* = 183.56	*F*(000) = 376
Orthorhombic, *C**m**c*2_1_	*D*_x_ = 1.753 Mg m^−^^3^
Hall symbol: C 2c -2	Mo *K*α radiation, λ = 0.71073 Å
*a* = 6.6807 (17) Å	µ = 0.52 mm^−^^1^
*b* = 10.4409 (17) Å	*T* = 293 K
*c* = 9.9707 (19) Å	Block, colourless
*V* = 695.5 (2) Å^3^	0.35 × 0.34 × 0.30 mm

### Data collection

**Table:**

Bruker SMART APEXII CCD area-detector diffractometer	708 independent reflections
Radiation source: fine-focus sealed tube	696 reflections with *I* > 2σ(*I*)
Graphite monochromator	*R*_int_ = 0.019
phi and ω scans	θ_max_ = 27.9°, θ_min_ = 3.6°
Absorption correction: multi-scan (*SADABS*; Sheldrick, 2000)	*h* = −8→8
*T*_min_ = 0.839, *T*_max_ = 0.860	*k* = −13→12
1968 measured reflections	*l* = −11→13

### Refinement

**Table:**

Refinement on *F*^2^	Hydrogen site location: inferred from neighbouring sites
Least-squares matrix: full	H-atom parameters constrained
*R*[*F*^2^ > 2σ(*F*^2^)] = 0.021	*w* = 1/[σ^2^(*F*_o_^2^) + (0.0313*P*)^2^ + 0.199*P*] where *P* = (*F*_o_^2^ + 2*F*_c_^2^)/3
*wR*(*F*^2^) = 0.055	(Δ/σ)_max_ < 0.001
*S* = 1.10	Δρ_max_ = 0.18 e Å^−^^3^
708 reflections	Δρ_min_ = −0.16 e Å^−^^3^
62 parameters	Extinction correction: *SHELXL97* (Sheldrick, 2008), Fc^*^=kFc[1+0.001xFc^2^λ^3^/sin(2θ)]^-1/4^
1 restraint	Extinction coefficient: 0.227 (9)
Primary atom site location: structure-invariant direct methods	Absolute structure: Flack (1983), 252 Friedel pairs
Secondary atom site location: difference Fourier map	Flack parameter: 0.09 (7)

### Special details

**Table:**

Geometry. All e.s.d.'s (except the e.s.d. in the dihedral angle between two l.s. planes) are estimated using the full covariance matrix. The cell e.s.d.'s are taken into account individually in the estimation of e.s.d.'s in distances, angles and torsion angles; correlations between e.s.d.'s in cell parameters are only used when they are defined by crystal symmetry. An approximate (isotropic) treatment of cell e.s.d.'s is used for estimating e.s.d.'s involving l.s. planes.
Refinement. Refinement of *F*^2^ against ALL reflections. The weighted *R*-factor *wR* and goodness of fit *S* are based on *F*^2^, conventional *R*-factors *R* are based on *F*, with *F* set to zero for negative *F*^2^. The threshold expression of *F*^2^ > σ(*F*^2^) is used only for calculating *R*-factors(gt) *etc*. and is not relevant to the choice of reflections for refinement. *R*-factors based on *F*^2^ are statistically about twice as large as those based on *F*, and *R*- factors based on ALL data will be even larger.

### Fractional atomic coordinates and isotropic or equivalent isotropic displacement parameters (Å^2^)

**Table:**

	*x*	*y*	*z*	*U*_iso_*/*U*_eq_	
Cl	0.0000	0.34257 (4)	0.24339 (6)	0.03211 (19)	
N2	0.0000	0.14030 (15)	0.5026 (2)	0.0277 (4)	
O3	0.0000	0.07128 (19)	0.7449 (3)	0.0571 (6)	
C1	0.1613 (2)	0.35120 (12)	0.57848 (17)	0.0255 (3)	
H1A	0.2305	0.3773	0.6596	0.031*	
H1B	0.2557	0.3293	0.5081	0.031*	
N3	0.0000	0.1881 (2)	0.7388 (3)	0.0354 (4)	
N1	0.0000	0.44299 (15)	0.5346 (2)	0.0269 (4)	
H1C	0.0000	0.4581	0.4457	0.032*	
H1D	0.0000	0.5170	0.5808	0.032*	
O4	0.0000	0.2614 (3)	0.8318 (2)	0.0536 (6)	
O1	0.16253 (18)	0.10036 (10)	0.46749 (16)	0.0425 (4)	
C2	0.0000	0.24966 (18)	0.6017 (2)	0.0217 (4)	

### Atomic displacement parameters (Å^2^)

**Table:**

	*U*^11^	*U*^22^	*U*^33^	*U*^12^	*U*^13^	*U*^23^
Cl	0.0359 (3)	0.0347 (3)	0.0258 (3)	0.000	0.000	0.0048 (3)
N2	0.0314 (9)	0.0207 (8)	0.0309 (11)	0.000	0.000	0.0002 (7)
O3	0.0562 (12)	0.0522 (10)	0.0629 (14)	0.000	0.000	0.0373 (12)
C1	0.0226 (7)	0.0236 (7)	0.0304 (8)	−0.0015 (5)	0.0008 (6)	−0.0013 (5)
N3	0.0232 (8)	0.0556 (11)	0.0273 (9)	0.000	0.000	0.0133 (13)
N1	0.0323 (10)	0.0202 (7)	0.0283 (9)	0.000	0.000	−0.0012 (7)
O4	0.0463 (12)	0.0904 (16)	0.0240 (9)	0.000	0.000	−0.0017 (9)
O1	0.0365 (7)	0.0362 (6)	0.0549 (9)	0.0117 (4)	−0.0002 (7)	−0.0136 (6)
C2	0.0218 (9)	0.0215 (9)	0.0217 (11)	0.000	0.000	0.0007 (7)

### Geometric parameters (Å, º)

**Table:**

N2—O1^i^	1.2147 (14)	C1—H1B	0.9700
N2—O1	1.2147 (14)	N3—O4	1.203 (4)
N2—C2	1.510 (3)	N3—C2	1.511 (3)
O3—N3	1.221 (3)	N1—C1^i^	1.5073 (19)
C1—N1	1.5073 (18)	N1—H1C	0.9000
C1—C2	1.5294 (19)	N1—H1D	0.9000
C1—H1A	0.9700	C2—C1^i^	1.5294 (19)
			
O1^i^—N2—O1	126.74 (19)	O4—N3—C2	115.2 (2)
O1^i^—N2—C2	116.63 (9)	O3—N3—C2	118.0 (3)
O1—N2—C2	116.63 (9)	C1—N1—C1^i^	91.30 (15)
N1—C1—C2	88.89 (11)	C1—N1—H1C	113.4
N1—C1—H1A	113.8	C1—N1—H1D	113.4
C2—C1—H1A	113.8	H1C—N1—H1D	110.7
N1—C1—H1B	113.8	N2—C2—N3	105.67 (17)
C2—C1—H1B	113.8	N2—C2—C1	115.17 (13)
H1A—C1—H1B	111.1	N3—C2—C1	115.58 (13)
O4—N3—O3	126.7 (3)	C1—C2—C1^i^	89.62 (15)
			
C2—C1—N1—C1^i^	8.66 (17)	O3—N3—C2—N2	0.0
O1^i^—N2—C2—N3	89.58 (16)	O4—N3—C2—C1	51.39 (11)
O1—N2—C2—N3	−89.58 (16)	O3—N3—C2—C1	−128.61 (11)
O1^i^—N2—C2—C1	−141.56 (16)	O4—N3—C2—C1^i^	−51.39 (11)
O1—N2—C2—C1	39.3 (2)	O3—N3—C2—C1^i^	128.61 (11)
O1^i^—N2—C2—C1^i^	−39.3 (2)	N1—C1—C2—N2	109.30 (15)
O1—N2—C2—C1^i^	141.56 (16)	N1—C1—C2—N3	−126.93 (15)
O4—N3—C2—N2	180.0	N1—C1—C2—C1^i^	−8.53 (17)

Symmetry code: (i) −*x*, *y*, *z*.

### Hydrogen-bond geometry (Å, º)

**Table:**

*D*—H···*A*	*D*—H	H···*A*	*D*···*A*	*D*—H···*A*
N1—H1*C*···Cl	0.90	2.35	3.087 (2)	139
N1—H1*D*···Cl^ii^	0.90	2.19	3.0575 (19)	163
C1—H1*B*···O1	0.97	2.50	2.8432 (19)	100
C1—H1*B*···O4^iii^	0.97	2.58	3.543 (2)	172

Symmetry codes: (ii) −*x*, −*y*+1, *z*+1/2; (iii) *x*+1/2, −*y*+1/2, *z*−1/2.
